# Use of the Blue Button Online Tool for Sharing Health Information: Qualitative Interviews With Patients and Providers

**DOI:** 10.2196/jmir.4595

**Published:** 2015-08-18

**Authors:** Dawn M Klein, Gemmae M Fix, Timothy P Hogan, Steven R Simon, Kim M Nazi, Carolyn L Turvey

**Affiliations:** ^1^ Iowa City VA Health Care System Comprehensive Access and Delivery Research and Evaluation (CADRE) Center Iowa City, IA United States; ^2^ University of Iowa Carver College of Medicine Department of Psychiatry Iowa City, IA United States; ^3^ Edith Nourse Rogers Memorial Veterans Hospital Center for Healthcare Organization and Implementation Research (CHOIR), A VA HSR&D Center of Innovation Bedford, MA United States; ^4^ Boston University School of Public Health Department of Health Policy and Management Boston, MA United States; ^5^ University of Massachusetts Medical School Division of Health Informatics and Implementation Science Department of Quantitative Health Sciences, Worcester, MA United States; ^6^ Edith Nourse Rogers Memorial Veterans Hospital eHealth Quality Enhancement Research Initiative, National eHealth QUERI Coordinating Center Bedford, MA United States; ^7^ Veterans Affairs Boston Healthcare System Section of General Internal Medicine Boston, MA United States; ^8^ Brigham and Women’s Hospital Division of General Medicine and Primary Care Boston, MA United States; ^9^ Veterans Health Administration Veterans and Consumers Health Informatics Office Office of Informatics & Analytics Washington, DC United States

**Keywords:** health record, personal, consumer health information technology, care coordination, qualitative research, eHealth, health care providers, information sharing, meaningful use

## Abstract

**Background:**

Information sharing between providers is critical for care coordination, especially in health systems such as the United States Department of Veterans Affairs (VA), where many patients also receive care from other health care organizations. Patients can facilitate this sharing by using the Blue Button, an online tool that promotes patients’ ability to view, print, and download their health records.

**Objective:**

The aim of this study was to characterize (1) patients’ use of Blue Button, an online information-sharing tool in VA’s patient portal, My Health*e*Vet, (2) information-sharing practices between VA and non-VA providers, and (3) how providers and patients use a printed Blue Button report during a clinical visit.

**Methods:**

Semistructured qualitative interviews were conducted with 34 VA patients, 10 VA providers, and 9 non-VA providers. Interviews focused on patients’ use of Blue Button, information-sharing practices between VA and non-VA providers, and how patients and providers use a printed Blue Button report during a clinical visit. Qualitative themes were identified through iterative rounds of coding starting with an a priori schema based on technology adoption theory.

**Results:**

Information sharing between VA and non-VA providers relied primarily on the patient. Patients most commonly used Blue Button to access and share VA laboratory results. Providers recognized the need for improved information sharing, valued the Blue Button printout, and expressed interest in a way to share information electronically across settings.

**Conclusions:**

Consumer-oriented technologies such as Blue Button can facilitate patients sharing health information with providers in other health care systems; however, more education is needed to inform patients of this use to facilitate care coordination. Additional research is needed to explore how personal health record documents, such as Blue Button reports, can be easily shared and incorporated into the clinical workflow of providers.

## Introduction

Care coordination can be defined as the deliberate organization of patient care activities among people involved in a patient’s care to facilitate the appropriate delivery of health care services [1]. The Institute of Medicine identified care coordination as one of 20 priorities to improve the quality of health care in the United States [2]. The accurate and timely sharing of information is a critical component for effective coordination. In 2013, a survey conducted by the Office of the National Coordinator revealed that 1 in 3 individuals reported a gap in information exchange when seeking care for a medical problem. In addition, half of those who experienced a gap reported they had to tell the provider about their medical history because records were not received by another provider [3]. When information is lacking, patient safety may be compromised, duplicate services received (ie, unnecessary repeat laboratory testing), and health care costs increased [4-10].

One challenge to effective information sharing is that medical records are controlled and managed by health care organizations often resulting in a fragmented record for patients who receive care from multiple health care systems [11]. Electronic health records (EHRs) and health information exchanges (HIEs) are organizational health information technology solutions that are intended to help improve communication within and between care settings [12,13]. Although there has been was significant progress in EHR implementation, adoption of HIEs is variable across states and organizations [14]. In a study by Furukawa et al [15], only 14% of providers stated that they exchanged health information electronically with providers practicing outside of their health care system.

In contrast, electronic personal health records (PHRs) are managed by individuals [16,17]. Patient PHRs (patient portals) tethered to EHRs can help patients access information easily. The ability to do so has increased in recent years, in part due to meaningful use criteria that require health care systems to provide patients with access to their own health information [18-20]. Stage 2 of the meaningful use criteria further expands this mandate with requirements to provide patients the ability to transmit information securely [21]. Consumer-mediated exchange complements organizational HIE between care providers and systems. Given the requirements for consumer access to their health information, many organizations have adopted the Blue Button [22]. However, little is known about patient and provider attitudes about consumer-mediated exchange.

The Blue Button concept emerged in January 2010 at a Markle Consumer Engagement Workgroup with the goal of empowering consumers by providing them the ability to generate and download a single electronic file that contains their personal health information [23]. In August 2010, the Department of Veterans Affairs (VA) launched their Blue Button in My Health*e*Vet, VA’s combined patient portal and PHR [24]. Blue Button allows patients to easily view, print, and download their VA medical record data and self-entered information to create a report of their health information. Patients are able to customize the information they choose to include in their report by date range and data class. To date, more than 500 payers, providers, health-related associations, and others have taken the Blue Button Pledge to promote patient access to their own health data [25]. Although Blue Button awareness has grown, there is still a significant need for education because one-third of providers in a 2014 survey reported no familiarity with the Blue Button initiative [26]. With growing consumer access to their health information, it is important to examine patients and providers perceptions and experiences with Blue Button adoption and how it may be used to improve care coordination.

Recipients of VA health care benefits routinely receive care from both VA and non-VA providers. The 2011 Survey of Veteran Enrollees’ Health and Reliance upon VA found that 77% of Veterans had alternative health insurance (ie, Medicare) [27]. In a study of rural Veterans, 75% indicated receiving care outside of the VA in the last year [28]. The 2014 Veterans Access, Choice, and Accountability Act [29] is also expected to increase care in civilian sectors making the need for effective care coordination even more critical for VA patients.

This study sought to understand how VA patients were adopting the Blue Button, information-sharing practices between VA and non-VA providers, and providers’ thoughts about patients as the mediators of HIE. Further, with Blue Button and consumer-mediated sharing of health information as newer concepts, this study explored (1) if a report printed using the Blue Button could facilitate information sharing to support clinical care and (2) patient and provider preferences regarding receipt of this information.

## Methods

### Study Design

Qualitative interviews were conducted in 2012 as part of a larger evaluation of Blue Button adoption and use [30]. The focus of this study was on patient and provider experiences with PHRs and specifically the My Health*e*Vet Blue Button feature to identify (1) barriers and facilitators to adoption of Blue Button and (2) use of the Blue Button printout to improve coordination of care among all members in a Veteran’s treatment team.

### Participants

A rural Midwest and an urban Northeast VA health care system participated to represent both rural and urban locations. VA patients registered with a My Health*e*Vet account were identified and invited to participate by letter. For select sampling, interested participants were asked about prior Blue Button use and if they received care from a non-VA provider. At the Midwest VA, non-VA providers were recruited through letter invitation from the local state association. Non-VA and VA providers were also identified with the assistance of project coinvestigators (CT, SS). Participants completed an informed consent process and all study procedures were approved by both Institutional Review Boards.

Using Rogers’ Diffusion of Innovation theory [31] and the Unified Theory of Acceptance and Use of Technology (UTAUT) [32] as models, researchers formed interview questions (see Textbox 1 for examples of interview questions). These theories provided a flexible framework for determining key components influencing patients’ adoption and use of Blue Button, and providers’ perspectives toward use of Blue Button and PHRs for information sharing. Interview questions addressed how participants learned about Blue Button (knowledge/awareness), experiences using Blue Button (ease of use), and whether information printed from Blue Button was brought to health care visits (implementation), etc. Patients were asked about communication between their providers and completed a baseline questionnaire about demographics and prior experience with My Health*e*Vet. Providers were asked about their experience coordinating care between organizations for VA patients, preferences for patients sharing information, and for input on the essential information to be included in a health summary. At the end of the interview, all participants were provided a sample 20-page Blue Button printout (Multimedia Appendix 1) based on a test patient with both VA EHR data (appointments, medication history, allergies, laboratory results, wellness reminders) and self-entered data (demographics, emergency contacts, health care providers, treatment facilities, health insurance, medications and supplements, allergies/adverse reactions, labs and tests, medical events, immunizations, vitals and readings, family health history, military health history). Participants critiqued the content and display and were asked for their opinions on using the document during a clinical visit. Interviews were conducted by trained research personnel primarily by phone and, when feasible, some provider interviews at the Midwest VA occurred in-person.

**Examples of semistructured interview questions for VA patients and providers.**

VA Patients

- How do your doctors coordinate your medical care? How do they communicate?
- Tell me a little bit about your experience using My Health*e*Vet. What do you know about the Blue Button? Tell me about your experience using Blue Button.
- What do you think of the format of the information in the printout? Is it easy to understand?
- Have you ever brought information that you printed from Blue Button to a health care visit?
- If you were able to delegate your access to your personal health record / My Health*e*Vet account to your health care provider would you? (Meaning would you give your doctor the ability to log in to your My Health*e*Vet account?)

Providers

- Tell me about your experience giving and receiving information from [VA or non-VA] providers about patients’ treatment (eg, medications).
- Are you familiar with personal health records / patient portals? Have you heard of the Blue Button?
- What is the essential information you want in a health summary? What do you think of the format of the Blue Button printout?
- Have you had experiences with patients who have brought in information from their personal health record, such as My Health*e*Vet?
- For patients who receive care from both VA and non-VA providers, what are your preferences on the best way for patients to share their health information with you?
- If a patient brought in a printout like this, what would you do with it?
- If a patient was able to delegate you access to their personal health record, do you think you would access it?

### Analysis

Interviews were audio recorded, manually transcribed verbatim, and entered into the qualitative data software, NVivo 8 (QSR International Pty Ltd, Victoria, Australia) for coding. Codes were developed a priori based on concepts from Diffusion of Innovation [31] and UTUAT [32] theories. Additional, emergent themes were identified through grounded thematic approaches [33]. The research team (GF, CT, DK) developed codebooks specific to each participant group: non-VA providers, VA providers, and patients. For each codebook, the team systematically reviewed 2 to 3 interviews together and discussed key concepts. Codebooks were iteratively developed until no new codes were identified and saturation was reached. For the provider interviews, fewer interviews were needed to reach saturation because their perspectives tended to be more similar whereas there was greater variability among patients. All interviews were then coded by a trained research assistant who worked closely with the team to consistently code the data. If there was clarification needed to address or revise codes, a team meeting was held to reach consensus.

## Results

### Participant Characteristics

A total of 34 patients completed interviews. See Table 1 for sample characteristics of participants. Of these, 24 VA patients reported using Blue Button previously and 22 received care from a non-VA provider in addition to their VA provider. In all, 19 providers (9 non-VA and 10 VA) participated. Of the 10 VA providers interviewed, all were medical doctors (3 family practice, 4 internal medicine, 2 specialty care, 1 hospitalist). Of the 9 non-VA providers, 2 were nurses and 7 were medical doctors (3 family practice, 4 internal medicine/ primary care).

**Table 1:** Patient characteristics (N=34).

Sample characteristics		Participants
Age (years), mean (SD)		61.6 (10.2)
Gender (male), n (%)		33 (97)
**Education, n (%)**		
	High school or some technical/college	20 (58)
	College graduate or more	14 (42)
**Income (US $; n=33), n (%)**		
	<$25,000	7 (21)
	$25,000-$50,000	13 (39)
	>$50,000	13 (39)
**How long registered for My Health*e*Vet, n (%)**		
	≤1 year	13 (38)
	2-3 years	15 (44)
	≥4 years	6 (18)
**How often use My Health*e*Vet, n (%)**		
	Less than once a month	11 (32)
	About once a month	16 (47)
	About once a week or more	7 (21)
**Comfort using My Health*e*Vet**		
	Very comfortable	13 (38)
	Somewhat comfortable	12 (35)
	Neither comfortable/uncomfortable	5 (15)
	Somewhat or very uncomfortable	4 (12)
**Self-rated health, n (%)**		
	Excellent or very good	13(38)
	Good	10 (29)
	Fair or poor	11 (32)

Seven themes were identified: (1) knowledge of Blue Button; (2) ease of use, content, and readability of the Blue Button printout; (3) relative advantage of using Blue Button to access and share VA information; (4) perceived value of Blue Button; (5) patient experiences sharing VA health information; (6) provider perspectives on workflow and data quality; and (7) preferences sharing and receiving information. Table 2 (patient) and Table 3 (providers) summarize these themes with illustrative quotes. Of note, several themes for clinicians were similar regardless of organizational affiliation and these are indicated with “all” in Table 3.

**Table 2:** Summary of themes by patient responses with illustrative quotes.

Theme		Patient quote
**Knowledge of Blue Button**		
	Found on their own exploring My Health*e*Vet	“...looking at My Health*e*Vet one day and then it just caught my eye...so I tried it.”
**Ease of use, content, and readability of Blue Button printout**		
	Mixed responses to the ease of use	“It’s easy to use and it’s self-explanatory.”
		“Frustrating.”
		“It’s complicated.”
	Can be too long in length	“What I don’t like is how much paper it takes up.”
**Relative advantage of Blue Button for accessing and sharing VA information**		
	Sharing information between providers relies on the patient	“...it’s up to me to move the information back and forth.”
	Online access is easy and saves time	“It’s easier than the phone and it’s a timesaver as well.”
		“It’s better than me sending a release to [VA administrative office] and having them mail me...I could just go online [to get results] instead of having to wait for this giant document to come.”
**Perceived value of Blue Button**		
	Engaged in their own health care	“...the first time I used it I was really happy because I was participating in my health care. I mean you can actually see real time what’s going on...which is really good. So it makes you part of the process.”
**Patient experience sharing VA health information**		
	Value in sharing information for time and money	“Saves a stick in the arm... saves them...the money and the time and effort...your lab results are just as good from 2 months ago, as they were from today to 3 weeks away.”
	Selective in what information is shared	“Typically just print the labs...here’s the copy of my VA lab work...”
**Preferences for sharing and receiving information**		
	Mixed response for preference to print or electronically share and preference based on what they perceive may be better for their provider	“I would prefer...[to] make the PDF from the Blue Button and then put it on my... iPad ...rather than printing off a bunch of paper.”
		“Just bring in a copy...it’s faster.”
		“For [provider] I’d prefer to give her the paper copy because of her time...that way she can look it over when she’s ready.”
	Value sharing self-reported information	“...with the over-the-counter medication and stuff like that they need to be aware of what was going on...the better treatment that I can get out of my provider is based on the more knowledge that they have, not out of an educated guess...”
	Supportive of delegate access to VA providers; mixed support for delegate access to non-VA providers	“I’m confident in my providers and know they would maintain proper amount of security and are very ethical health care providers.”
		“I don’t know how many...non-VA providers you want snoopin’ around in a VA record...I trust my doctor, but I don’t know, it’s a security type of thing with me.”

**Table 3:** Summary of themes by provider responses with illustrative quotes.

Theme		Provider quote
**Knowledge of Blue Button**		
	All: limited knowledge of Blue Button	“I’ve not seen that...this is not what I get from a VA patient. What I get from him is akin to an office visit summary.” (non-VA)
**Ease of use, content, and readability of Blue Button printout**		
	All: improve information display	“...it’s not that the information is not useful, it’s just that it’s displayed over too many pages.” (non-VA)
	Non-VA*:* useful information; missing patient VA visit information (last visit note)	“You’re not showing me the ‘patient visit’ here. The one thing that’s missing.” (non-VA)
**Relative advantage of Blue Button for accessing and sharing VA information**		
	All*:* sharing information often relies on the patient	“I will print out stuff and give it to the patient, I say, ‘Here, go give this to your urologist, okay?.’...and sometimes ...we tell the patients, ‘You could have anything you want sent to whomever you want. Just go out to the business desk and those folks will take care of it.’” (VA)
	Non-VA: difficult to get information from VA and patients sharing this information can help bridge the gap	“With VA, we get nothing...[W]e need something we have to call the VA or have the patient acquire it...Nothing is ever sent automatically from VA...and most of the time I don’t even know that they see the VA...” (non-VA)
		“...we haven’t had real good luck getting information from VA, so I think this is...better, the Blue Button.” (non-VA)
**Perceived value of Blue Button**		
	All: tool for patient education and value of self-report information	“Because people who are really reading or going through their records they are more involved in their health...they will learn more about their own health and their own medication.” (VA)
		“Having the self-report is important, because that allows you to figure out what you think is going on and what they think is going on is different. And bringing together different sources, like looking at their pill bottles...” (VA)
	Non-VA: improved efficacy	“...it would increase our accuracy and decrease our duplication of tests... it would make ...more economical sense for the patient insurance system as well.” (non-VA)
	Non-VA: abstract and incorporate relevant information in own electronic medical record	“It’s not just this sort of scanned PDF, but rather something that becomes useable and actionable.” (non-VA)
**Provider perspectives on workflow and data quality**		
	All: mixed response to how it would impact workflow of clinical encounter, however information was valued	“It would help...I don’t think it would add a lot of time...to have the information is important.” (non-VA)
		“If they’re very knowledgeable and could tell me all this verbally, then it probably doesn’t necessarily save time...if this was a very long printout, it could take longer, but I don’t think that’s necessarily a bad thing. Because if you you’re getting a fuller picture...then I think that would be beneficial.” (VA)
	All: generally trust Blue Button report and self-entered information; however, may depend on the data reported and patient	“We ask patients to give us their history of what’s happened to them, and we trust that. There are times we have to go corroborate that, so I see no reason why I wouldn’t trust this any less than my encounter when I ask, ‘So what’s happened in the last year?’” (non-VA)
		“[the self-entered information] I get a little nervous...did they enter right? ...it’s a data quality issue.” (VA)
**Preferences for sharing and receiving information**		
	All: prefer electronic receipt of data and easy sharing between systems	“What would be really ideal is if there were an interface between the community and the VA system where if a patient gets lab work done at the VA, or diagnostic studies done at the VA, or a colonoscopy done at the VA, right? Then, that stuff would come in and integrate with my system.” (non-VA)
	All: hard copy could be used for patient education	“I’d like them to bring it in their hand...We make notes on it, it goes back home with them. It’s incredibly valuable for them to have stuff in their hands.” (VA)
	All: receipt of information in advance of clinical encounter	“It’s always nice to have it ahead of the visit... then you can review it before the visit and highlight the important things that you want to address...” (non-VA)
	All: mixed support for delegate access to patient’s PHR	“In certain cases, I would...depends on the patient... and their problems.” (VA)
		“...it spills on the wrong side of patients embracing responsibility for their health record. And therefore, it probably spills on the wrong side of where the liability is...” (non-VA)
	All: open to patients logging in and sharing their personal health record data at a visit; however, possible work flow and technology barriers	“It’s going to take some time, but chronic illness management requires some time. You need to take time and talk to people about these things during visits, and I think this would be a way of making it more efficient, not less...it’s a tradeoff, you would save time by not doing it, but I think that not doing it is not a good option.” (VA)
		“Right now, they can’t log in to our computer, that would have to change...but, even so, I think that would sort of bog you down...in the office.” (non-VA)

### Knowledge of Blue Button

Most patients learned of the Blue Button simply seeing it on the My Health*e*Vet website. At the time of the study, VA and non-VA providers were generally unaware of the term “Blue Button” and had limited experience with patients using PHRs to share information with them. Among those who had heard of Blue Button, one non-VA provider did not know any details about it and a VA provider knew that patients could print a report using Blue Button, but had not actually received a printout from a patient.

### Ease of Use, Content, and Readability of the Blue Button Printout

This theme focused on patients’ ease of downloading a Blue Button file, content, and readability of the report. Patients primarily taught themselves how to use it with no specific training and there was variability with experiences in the ease of using the Blue Button. They liked the convenience of accessing their information online and that it was consolidated in one document, albeit the length of the printout was a concern for some.

Provider interviews focused on the sample Blue Button printout, critiquing the content and display of information. Overall, non-VA providers found the content useful; however, it was noted that some changes in formatting may improve the document. However, non-VA providers acknowledged that having the information outweighed the inconvenience of what they perceived as a cumbersome document due to its length.

When asked about the most important information to include in the Blue Button printout to inform clinicians, non-VA providers wanted a current medication list, laboratory test results, wellness reminders, immunizations, and allergies. In addition, there was interest in having the clinical note from the last visit available. VA providers reported much of the content redundant with information in the VA medical record and concurred with non-VA providers that the presentation of information could be improved.

### Relative Advantage of Using Blue Button to Access and Share Information

For patients, this theme emerged in their discussion of the advantage of online access to their health information. This access could then facilitate information sharing. All interviewees (patients, VA providers, and non-VA providers) indicated that communication between providers primarily relies on the patient. Non-VA providers reported great difficulty getting health information from the VA and saw Blue Button as a possible solution to this problem. One non-VA provider, after viewing the sample printout, reported a preference for the Blue Button printout over other records from VA. VA providers already have access to much of the information in the Blue Button, so the relative advantage of Blue Button for information sharing was not evident. In contrast, non-VA providers who struggled to access VA information saw clear value.

### Perceived Value of Blue Button

Two themes emerged under this domain: patient engagement and use of the Blue Button printout for clinical care and the health care system. With regards to patient engagement, patients liked having access to their health information. VA and non-VA providers reinforced this and expressed use of the printout as a tool that could help to identify gaps in understanding. Being better informed of all care can, in turn, help prevent errors or medication/test duplication and provide benefit from a cost perspective.

Non-VA providers were positive about the utility of the information for clinical decision making and indicated they would incorporate the information within their own medical records. One provider detailed the data sections (ie, allergies, medications) that he would integrate as structured data in their electronic record. In contrast to non-VA providers, VA providers saw little value in the printout for VA health information because it would be redundant with information already accessible in VA’s EHR. However, there was interest in reviewing the information that patient had self-entered into their PHR.

### Patient Experiences Sharing Health Information From Blue Button

Patients reported mainly using the Blue Button for their own knowledge and most often reviewed laboratory results and appointments. There was limited experience sharing it with providers; however, for those who did share their Blue Button information with a non-VA provider, it was most often laboratory results. Those Veterans who have used the Blue Button for sharing information, tended to only print specific data rather than a long report. Most reported a favorable response sharing it, but one patient did have a negative experience that resulted in his perception that “nobody wants this...a doctor wants their own opinion.”

### Provider Perspectives on Workflow and Data Quality

The non-VA and VA providers interviewed had little to no experience with patients sharing a Blue Button printout; thus, to address the concept of using this document for clinical care, providers were asked for their perspectives on workflow and time burden if a patient were to present with a Blue Button printout at a visit. Results were mixed. Some expressed it would add time with others indicating it would be a time saver. Overall, providers expressed that having the information outweighed concerns about added workload and time.

There was also discussion if providers would “trust” a Blue Button document received from a patient and self-reported information in the report. Many providers indicated they would trust the accuracy of information accessed using Blue Button. In regards to patient self-reported information, some added the stipulation that similar to any self-report of information, it may depend on the patient and type of information reported. However, a few providers did note concern related to data quality for self-report.

### Preferences for Sharing and Receiving Information

All patient and provider interviewees were also asked questions to explore their preferences for how information is shared to inform future guidance to VA patients using the Blue Button; this included receiving a printed hard copy either before or during a medical visit, electronic receipt, delegate access (assigning permission to allow the provider to sign into the patient’s My Health*e*Vet account), or the patient logging in to their My Health*e*Vet account during an appointment.

Patients tended to want to share their information in a way that would be convenient for the provider. The majority were comfortable delegating My Health*e*Vet access to their VA providers, but some indicated it would be duplicative for VA information. Despite this, they saw value in sharing their self-entered data. For delegating access to non-VA providers, there was not consensus and patients expressed conflicting views. One patient who noted the value of self-entered data and stated he would delegate access to his VA provider reported that he would not delegate access to his non-VA provider due to privacy concerns about sensitive health information. However, he would share a hard copy printout of selected information from his VA medical record if he deemed it necessary.

Patients who favored electronic information sharing (including logging in at an appointment to access their Blue Button) wanted to avoid unnecessary printing or misplacing of the document. Those who favored printing indicated that it was easier or faster.

Non-VA and VA providers expressed a desire for electronic receipt of information so that ideally it could be integrated into their EHR. Although it was often the preference, there was awareness that the exchange needed to be secure and a perception that this was not yet feasible with current technology. Some providers also wanted to receive the information in advance of an appointment. For others, hard copies shared during a visit were viewed as easier and one VA provider thought it could be used as a tool for patient education.

Provider support among both provider groups for delegate access was split. A non-VA provider agreed he would access the patient’s My Health*e*Vet account if authorized and a VA provider also supported access depending on the patient situation. Alternatively, time and liability were significant concerns. Providers seemed more open to having a patient log in to their PHR during an appointment; however, workflow barriers included time and lack of computer access in exam rooms.

## Discussion

In this sample of patients, VA providers, and non-VA providers, information sharing between providers relied primarily on the patient. Reports generated by the Blue Button feature of a PHR portal that contain both EHR data and patient self-entered information have great potential to facilitate care coordination in such contexts. Patients indicated some usability issues with the My Health*e*Vet Blue Button; however, they generally had favorable opinions of the technology. Providers recognized the need for improved information sharing. In particular, non-VA providers felt that having access to more VA health information would be of significant benefit. Many providers we spoke with expressed interest in a way to share information electronically across health care delivery settings. Although this study focused on VA patients receiving care from non-VA providers, meaningful use Stage 2 criteria promotes comparable consumer-mediated health information sharing between all health care settings.

These findings are consistent with other studies that found patients are responsible for sharing health information between providers [30,34-36] and limited use of the Blue Button printout despite its potential to improve information sharing [30]. The review of a sample Blue Button printout provided valuable insight about the potential impact of patients sharing their health information during a clinical encounter. Although the length of the report and time to review were potential barriers, in practice, patients who shared information using the Blue Button tended to be selective in choosing what they provided to their non-VA providers, consistent with other research [36,37]. Patients indicated they want to share information specifically relevant to their care and in a way convenient to the provider. One concern is that patients may not know exactly what information to share. Although this may be perceived in some cases as patients intentionally omitting what might be clinically relevant information, it may in fact be a function of health literacy. Patient portals provide easier and timelier access to the EHR data in an unprecedented way. However, as noted earlier, because medical information has historically been managed at an institutional level, patients may have limited experience managing their own health information. This study and the prior literature suggest both a need to educate patients about using their patient portal to share information and for patient portals to be designed in ways that support patients’ need to easily share critically important clinical information. In addition, it is equally important to inform providers about Blue Button and for providers to encourage patients to share their health data from providers in other care settings [38].

Non-VA provider experiences with care coordination are consistent with findings by Nayar et al [34]: 71% of non-VA providers surveyed reported they were rarely or never informed about VA care visits. Difficulty sharing information back to the VA was also apparent, with only 33% reporting that they had shared information with VA. HIEs are one mechanism that may help facilitate bidirectional exchange between systems [39], but adoption of HIEs is variable across states and organizations [14]. It is also critical for EHRs to support care coordination; however, EHRs often do not contain much information about outside care [40] and it is difficult to share across settings due to interoperability issues [12,41]. This supports the need for consumer-mediated information sharing because patients know where and when they will be receiving care. It also can empower patients by allowing them to choose what information to share and to be an engaged participant in their health care team.

This study provides support that providers and patients value and trust patient sharing of health record information and patient-generated data. Although patients can self-enter information in My Health*e*Vet, this information is not yet accessible to their VA providers. Data available from self-report in PHRs, remote monitoring devices, or personal wearables, can help inform providers’ understanding of a patient’s health between visits. Interest in these data is growing and it is important to develop meaningful ways for this data to be shared and integrated into workflow to help inform clinical care when relevant [42]. Efforts are underway for this information to be accessible to health care providers within the VA clinical information system and incorporated into clinical workflow.

Since these interviews, there have been continued enhancements to My Health*e*Vet, including increased patient access to information. In January 2013, clinical care notes were added to the Blue Button (VA OpenNotes) [43] and a Continuity of Care Document (CCD) / VA Health Summary became available. This document is a health summary that follows standards for interoperability with the goal of being integrated into other systems’ EHRs. The VA CCD includes many of the essential data specified by non-VA providers; however, it does not yet provide the most recent clinic note, which was requested by non-VA providers in this study. The ability for patients to securely transmit their CCD to an approved organization, non-VA provider, or application is currently in field testing.

Provider-focused HIE and consumer-mediated HIE can complement each other and provide benefit in different use cases. For example, in emergency department care, the immediate need for information may be served best by query or direct-based HIE models; whereas, in outpatient chronic care management, in which patients are often seen by multiple providers in different settings, consumer-mediated HIE can also effectively support care coordination. It is critical to educate patients about opportunities to participate in all types of HIE so they can make informed decisions about their preferences for sharing of their health information. As reflected by some participants in this study, some patients may not want to share all their health information with all providers. Expanding efforts in these areas supports the National Quality Strategy that focuses on patient engagement and effective communication as priorities to help meet the 3 broad aims of better, more affordable care for individuals and communities [44]. In a complex health care environment, multiple strategies are necessary to ensure information is available to provide safe and improved health care regardless of how the information is exchanged.

Limitations of this study include interviewing only patients who currently used My Health*e*Vet. Participants were asked to hypothesize how they would use the Blue Button printout for information sharing and clinical care, but it is unknown what they might do in actual practice. However, there is promising research reporting positive benefits of PHR use and patient engagement for health outcomes [45-49]. In addition, it was not required that patients have a non-VA provider; nevertheless, their use of the Blue Button to share self-reported information back to VA was relevant. The patients were predominantly older, white, male Veterans; younger or female patients may have different perspectives.

Although this study focused on the VA health care system, the results may be transferable to other settings where patients receive care from multiple health care systems and other organizations using Blue Button. In speaking to patients, VA providers, and non-VA providers, we gathered the perspectives from a range of stakeholders who are engaged in information sharing and use of tools such as Blue Button. Participants from geographically different regions of the country and different kinds of provider expertise (ie, family medicine, specialty care, and nursing) were included. In addition, meaningful diversity is also evident in the patient sample (ie, income, self-reported health status, and My Health*e*Vet experience). This variety of perspectives increases transferability [50] of findings across contexts.

More research is needed to examine whether patients sharing their health information with providers from different systems improves health care processes and outcomes. For example, does this consumer-mediated sharing improve medication reconciliation, reduce therapeutic duplication of medications, and/or reduce duplicate laboratory services or costs? In addition, future research should examine the impact on workflow for the provider receiving the information and, as transmit requirements from meaningful use 2 are implemented, examine how information is received and incorporated into the EHR of the receiving provider or organization. As technology advances, effective processes must be developed in all care settings to enable all providers engaged in a patient’s care to effectively share information for care coordination.

## Appendix

Multimedia Appendix 1 Sample Blue Button report.

## Abbreviations

CADRE: Comprehensive Access and Delivery Research and Evaluation
CCD: Continuity of Care Document
EHR: electronic health records
HIE: health information exchanges
PHR: personal health records
VA: Veterans Affairs
