# Supplementary material for: Use of the Blue Button Online Tool for Sharing Health Information: Qualitative Interviews With Patients and Providers
Source: J Med Internet Res. 2015 Aug 18;17(8):e199. doi: 10.2196/jmir.4595 (PMC4736289; doi:10.2196/jmir.4595)
Supplement: Multimedia Appendix 1 [file jmir_v17i8e199_app1.pdf]

MEDICAL CONFIDENTIAL

My HealtheVet

Personal Information Report

*************CONFIDENTIAL*************

Produced by the VA Blue Button (v12)

06 Dec 2011 @ 0736

This summary is a copy of information from your My HealtheVet Personal Health Record. Your summary contains information that you entered and may also include a copy of some of the information in your VA medical record as it becomes available in My HealtheVet. Please let your health care team know if you have questions about your health information. For some Veterans, information from the Department of Defense (DoD) is available. You will see this in VA Blue Button as DoD Military Service Information.

Name: MHVVETERAN, ONE A

Date of Birth: 01 Mar 1948

Demographics

Source:	Self-Entered
First Name:	ONE
Middle Initial:	A
Last Name:	MHVVETERAN
Suffix:	
Alias:	MHVVET
Relationship to VA:	Patient, Veteran, Employee
Current Occupation	Truck Driver
Home Phone Number:	000-010-0101
Work Phone Number:	000-020-0202
Pager Number:	000-030-0303
Cell Phone Number:	000-040-0404
FAX Number:	000-050-0505

Date of Birth:	01 Mar 1948
Gender:	Male
Blood Type:	AB+
Organ Donor:	Yes
Marital Status:	Married

Mailing or Destination Address:
123 Anywhere Road
Apartment 123
Anywhere, DC
United States
00001

Email Address:	mhvveteran@emailaddress.com
Preferred Method of Contact:	Email

MEDICAL CONFIDENTIAL
Blue Button Download My Data

Emergency Contacts

Contact First Name:	Two	Mailing Address:
Contact Last Name:	MHVVeteran	123 Anywhere Road<br>Apartment 123<br>Anywhere, DC<br>United States<br>00001
Relationship:		
Home Phone Number:	000-010-0101	
Work Phone Number:	000-060-0606 Ext 123	
Cell Phone Number:	000-070-0707	
Email:	twomhvveteran@domain.com	

Contact First Name:	Three	Mailing Address:
Contact Last Name:	MHVVeteran	123 Anywhere Road<br>Apartment 123<br>Anywhere, DC<br>United States<br>00001
Relationship:		
Home Phone Number:	000-010-0101	
Work Phone Number:	000-080-0808 Ext 123	
Cell Phone Number:	000-090-0909	
Email:	threemhvveteran@domain.com	

MEDICAL CONFIDENTIAL

Healthcare Providers

Source:	Self-Entered

Provider Name:	John Doe
Type of Provider:	Primary
Other Clinician Information:	
Phone Number:	000-000-0000 Ext: 1234
Email:	provider@institution.org
Comments:	Dr. Doe can be reached on the weekend if needed by leaving a message with the clinic.

Provider Name:	Jane Smith
Type of Provider:	Specialist
Other Clinician Information:	Seen as needed
Phone Number:	000-000-0001 Ext: 1234
Email:	specialist@institution.org
Comments:	Dr. Smith should be notified of any changes in my medical condition. Requires a referral from my health insurance company.

Blue Button Download My Data SM
MEDICAL CONFIDENTIAL

Treatment Facilities

Source:	Self-Entered

Facility Name:	Anywhere VA Medical Center	
Facility Type:	VA	Mailing Address:
VA Home Facility:	Yes	123 VA Drive<br>Suite 4<br>Anywhere, DC<br>United States<br>00001
Phone Number:	000-000-0001 Ext:1234	
FAX Number:	000-000-0002	
Comments:	Contact clinic when calling to make my appointments.	

Facility Name:	Health Care Inc.	
Facility Type:	Non-VA	Mailing Address:
VA Home Facility:	No	123 Anywhere Road<br>B-Wing<br>Anywhere, DC<br>United States<br>00001
Phone Number:	000-000-0002 Ext:5678	
FAX Number:	000-000-0003	
Comments:	Reminder to bring My HealtheVet printouts for all visits	

Blue Button Download My Data SM
MEDICAL CONFIDENTIAL

Health Insurance

Source:	Self-Entered

Health Insurance Company:	My Health Insurance Company		
ID Number:	0001234	Group Number:	0000000
Primary Insurance Provider:	Yes	Start Date:	01 Jan 2000
Insured:	One Mhvveteran	Stop Date:	
Pre-Approval Phone Number:	000-000-0003		
Health Insurance Company Phone Number:	000-000-0004		
Comments:	Need to get pre-authorization for special services.		

Health Insurance Company:	My Other Health Insurance Company		
ID Number:	000567891010	Group Number:	ABC123456789
Primary Insurance Provider:	No	Start Date:	01 Jan 2009
Insured:	One Mhvveteran	Stop Date:	
Pre-Approval Phone Number:	000-000-0005		
Health Insurance Company Phone Number:	000-000-0005		
Comments:	Coverage only for vision and dental.		

Blue Button Download My Data
MEDICAL CONFIDENTIAL

VA Wellness Reminders

Source:	VA
Last Updated:	06 Dec 2011 @ 0735
Sorted By:	Name (Ascending)

Learn more about these Wellness Reminders by visiting My HealtheVet. Please contact your health care team with any questions about your VA Wellness Reminders.

Wellness Reminder	Due Date	Last Completed	Location
Influenza Vaccine	DUE NOW	UNKNOWN	DAYT29
Colon Cancer Screening	DUE NOW	UNKNOWN	DAYT29
FYI-More Personal Wellness Info	DUE NOW	UNKNOWN	DAYT29

MEDICAL CONFIDENTIAL
Blue Button Download My Data

VA Appointments

Source:	VA
Last Updated:	06 Dec 2011 @ 0735
Sorted By:	Date (Descending)
VA Past Appointments are limited to two years from the date of your download request.	

Past Appointments

Date/Time:	13 Oct 2011 @ 1600
Location:	DAYT29 TEST LAB
Status:	NOT APPLICABLE
Clinic:	C&P XXXXXX
Phone Number:	3929
Type:	Compensation and Pension Appointment

Date/Time:	07 Sep 2011 @ 1100
Location:	DAYT29 TEST LAB
Status:	NOT APPLICABLE
Clinic:	TELEPHONE CALLS/GERIATRICS
Phone Number:	XXXX

Date/Time:	27 Jul 2011 @ 1400
Location:	DAYT29 TEST LAB
Status:	NOT APPLICABLE
Clinic:	DIABETIC-XXXXXX
Phone Number:	800-123-1234
Note:	This Appointment has preappointment activity scheduled:<br>Lab: 27 Jul 2011 @ 1000

Date/Time:	15 Jun 2011 @ 1300
Location:	DAYT29 TEST LAB
Status:	NOT APPLICABLE
Clinic:	DIABETIC-XXXXXX
Phone Number:	800-123-1234
Note:	This Appointment has preappointment activity scheduled:<br>Lab: 15 Jun 2011 @ 0800<br>EKG: 15 Jun 2011 @ 1030<br>X-Ray: 15 Jun 2011 @ 0900

Date/Time:	03 May 2011 @ 1100
Location:	DAYT29 TEST LAB
Status:	NOT APPLICABLE
Clinic:	TELEPHONE CALLS/GERIATRICS

MEDICAL CONFIDENTIAL

VA Medication History

Source:	VA
Last Updated:	11 Apr 2011 @ 1737
Sorted By:	Last Filled On (Descending)

VA Medication History includes up to two years of medication history unless you select a different date range in your download request.

Medication:	AMLODIPINE BESYLATE 10MG TAB
Instructions:	TAKE ONE TABLET BY MOUTH TAKE ONE-HALF TABLET FOR 1 DAY --AVOID GRAPEFRUIT JUICE--
Status:	Active
Refills Remaining:	3
Last Filled On:	20 Aug 2010
Initially Ordered On:	13 Aug 2010

Quantity	Days Supply	Pharmacy	Prescription Number
45	90	DAYTON	2718953

Medication:	IBUPROFEN 600MG TAB
Instructions:	TAKE ONE TABLET BY MOUTH FOUR TIMES A DAY WITH FOOD AS NEEDED
Status:	Active
Refills Remaining:	3
Last Filled On:	20 Aug 2010
Initially Ordered On:	01 Jul 2010

Quantity	Days Supply	Pharmacy	Prescription Number
240	60	DAYTON	2718960

Medication:	INSULIN,GLARGINE,HUMAN 100 UNT/ML INJ
Instructions:	INJECT 10 ML VIAL UNDER THE SKIN AS DIRECTED FOR 28 DAYS INJECT 25 UNITS UNDER THE SKIN AT BEDTIME DO NOT MIX WITH OTHER INSULINS/DISCARD OPEN VIALS AFTER 28 DAYS
Status:	Active
Refills Remaining:	3
Last Filled On:	20 Aug 2010
Initially Ordered On:	01 Jul 2010

Quantity	Days Supply	Pharmacy	Prescription Number
30	30	DAYTON	2718956

Medication:	TERAZOSIN HCL 2MG CAP
Instructions:	TAKE THREE CAPSULES BY MOUTH AT BEDTIME.
Status:	Active
Refills Remaining:	2
Last Filled On:	20 Aug 2010

MEDICAL CONFIDENTIAL

Medications & Supplements

Source:	Self-Entered		

Category:	RX Medication		
Drug Name:	Aspirin EC		
Prescription Number:	010101B	Start Date:	15 Jun 2005
Strength:	81mg	Stop Date:	
Dose:	1 tab		
Frequency:	daily		
Pharmacy Name:	My Local Drugstore		
Pharmacy Phone:	000-010-0000		
Reason for Taking:	Daily regimen for heart health		
Comments:			

Category:	OTC		
Drug Name:	Cough Medicine		
Prescription Number:		Start Date:	01 Feb 2010
Strength:	1000mg	Stop Date:	21 Feb 2010
Dose:	2 TBS		
Frequency:	morning and night		
Pharmacy Name:	My Local Drugstore		
Pharmacy Phone:	000-010-0000		
Reason for Taking:	Cough was keeping me up at night		
Comments:	Cleared up after a few weeks		

Category:	Herbal		
Drug Name:	Ginkgo biloba		
Prescription Number:		Start Date:	08 Mar 2008
Strength:		Stop Date:	
Dose:	2 capsules		
Frequency:	once a day		
Pharmacy Name:	My Local Drugstore		
Pharmacy Phone:	000-010-0000		
Reason for Taking:	Wife suggested to improve concentration		
Comments:	Take in the morning with breakfast		

Category:	Supplement		
Drug Name:	Multi-vitamin		
Prescription Number:		Start Date:	18 Mar 2010
Strength:	100% RDA	Stop Date:	
Dose:	1 tablet daily		
Frequency:	morning		

MEDICAL CONFIDENTIAL

VA Allergies

Source:	VA
Last Updated:	06 Dec 2011 @ 0735
Sorted By:	Date (Descending)
Please contact your health care team with any questions about your VA Allergy information.	

Allergy Name:	TRIMETHOPRIM	Date Entered:	09 Mar 2011
Allergy Type:	DRUG	Location:	DAYT29
Reaction:			
VA Drug Class:	ANTI-INFECTIVES,OTHER		
Observed/Historical:	HISTORICAL		
Comments:	The reaction to this allergy was MILD (NO SQUELAE)		

Allergy Name:	TRAMADOL	Date Entered:	09 Mar 2011
Allergy Type:	DRUG	Location:	DAYT29
Reaction:	URINARY RETENTION		
VA Drug Class:	NON-OPIOID ANALGESICS		
Observed/Historical:	HISTORICAL		
Comments:	gradually worsening difficulty emptying bladder -- might try tramadol again cautiously because pt. reported pain releif		

Allergy Name:	BACTRIM	Date Entered:	09 Mar 2011
Allergy Type:	DRUG	Location:	DAYT29
Reaction:			
VA Drug Class:	SULFONAMIDE/RELATED ANTIMICROBIALS		
Observed/Historical:	HISTORICAL		
Comments:	Causes Swelling of the Extremeties		

Allergy Name:	TERAZOSIN	Date Entered:	09 Mar 2011
Allergy Type:	DRUG	Location:	DAYT29
Reaction:	DIZZINESS		
VA Drug Class:	ALPHA BLOCKERS/RELATED		
Observed/Historical:	HISTORICAL		
Comments:	Lightheadedness on 10mg qhs Terazoin resolved upon stopping Terazoin.		

Allergy Name:	METHOCARBAMOL	Date Entered:	09 Mar 2011
Allergy Type:	DRUG	Location:	DAYT29
Reaction:	CONFUSION, DROWSINESS		
VA Drug Class:	SKELETAL MUSCLE RELAXANTS		
Observed/Historical:	OBSERVED		
Comments:	Drowsiness and hallucinations while on methocarbamol plus carbamazepine and other sedatives		

MEDICAL CONFIDENTIAL

Allergies/Adverse Reactions

Source:	Self-Entered

Allergy Name:	Peanuts	Date:	01 Aug 1980
Severity:	Moderate	Diagnosed:	Yes
Reaction:	Hives		
Comments:	Avoid peanuts and peanut based foods. Foods cooked with peanut oil also cause the reaction		

Allergy Name:	Pollen	Date:	18 Mar 2010
Severity:	Mild	Diagnosed:	Yes
Reaction:	Watery eyes, itchy nose		
Comments:	Took an over the counter antihistamine		

Blue Button Download My Data SM
MEDICAL CONFIDENTIAL

Medical Events

Source:	Self-Entered

Medical Events:	Colonoscopy	Start Date:	18 Mar 2000
Response:	Colonoscopy when well	Stop Date:	18 Mar 2000
Comments:	Polyps were removed. Doctor said these were benign		

Medical Events:	Broken right arm	Start Date:	04 Jan 2010
Response:	Placed in cast from my hand to my elbow	Stop Date:	17 Feb 2010
Comments:	Went to community hospital emergency room since I was on vacation. Followed up<br>with my VA doctor when I returned home.		

MEDICAL CONFIDENTIAL
Blue Button Download My Data SM

Immunizations

Source:	Self-Entered

Immunization:	Measles + Rubella (German Measles)	Date Received:	01 Mar 1950
Other:	none	Method:	Injection
Reactions:	Pain		
Comments:	Received as a child		

Immunization:	Flu	Date Received:	01 Sep 2009
Other:	H1N1	Method:	Inhalant
Reactions:			
Comments:	Had no reaction		

Immunization:	Tetanus	Date Received:	18 Jul 2010
Other:	none	Method:	Injection
Reactions:	Pain		
Comments:	stepped on a rusty nail		

Blue Button Download My Data SM
MEDICAL CONFIDENTIAL

MEDICAL CONFIDENTIAL

VA Laboratory Results

Source:	VA
Last Updated:	06 Dec 2011 @ 0735
Sorted By:	Date Specimen Collected (Descending)

VA Laboratory Results includes up to two years of history unless you select a different date range in your download request. Test results slightly outside the reference range are not unusual. Your provider has reviewed your test results and will contact you for any important issues. If you have further questions, please do not hesitate to contact your primary care provider.

Lab Test:	Prothrombin Time+PTT		
Lab Type:	Chemistry/Hematology	Ordering Provider:	XXXXXX
Specimen:	Plasma	Ordering Location:	DAYTON, OH VAMC
Date/Time Collected:	27 Jun 2011		

Test Name	Result	Units	Reference Range	Status	Performing Location
COAGULATION SURFACE INDUCED.FACTOR SUBSTITUTION~IMMEDIATELY AFTER ADDITION OF NORMAL PLASMA	13.2 Low	SECONDS	(59.0-104.0)	Final	DAYTON, OH VAMC
Interpretation:	Current normal range = 20.3 -35.8 seconds.<br>Normal range prior to 7/5/02 was 25.3 - 40.4 sec.<br>Normal range prior to 2/26/98 was 26.1 - 43.0 sec.<br>Therapeutic range prior to 7/5/02 was 67.0 - 118.0 sec.<br>Therapeutic range prior to 2/26/98 was 72.0 - 126.0 sec.				
COAGULATION TISSUE FACTOR INDUCED	9.9 Low	SECONDS	(23.0-33.0)	Final	DAYTON, OH VAMC
Interpretation:	Current normal range = 10.8 - 13.7 seconds.<br>Normal range prior to 7/5/02 was 10.7 - 14.1 seconds.<br>INR should be used for therapeutic decisions.				
COAGULATION TISSUE FACTOR INDUCED.INR	.7		(2-3)	Final	DAYTON, OH VAMC
Interpretation:	FOR ADDITIONAL INFORMATION - CHECK INR UNDER "TEST DESCRIPTION INFO"				

Comments:	For Test: COAGULATION (PT & PTT)<br>ANTICOAGULANT: PT

Performing Location Name/Address:

DAYTON, OH VAMC 4100 W. THIRD STREET , DAYTON, OH 45428

Lab Test:	Hemogram V		
Lab Type:	Chemistry/Hematology	Ordering	XXXXXX

		Provider:	
Specimen:	Whole blood	Ordering Location:	DAYTON, OH VAMC
Date/Time Collected:	22 Mar 2011 @ 1000		

Test Name	Result	Units	Reference Range	Status	Performing Location
ERYTHROCYTE DISTRIBUTION WIDTH	40 High	%	(11.5-14.5)	Final	DAYTON, OH VAMC
ERYTHROCYTE MEAN CORPUSCULAR HEMOGLOBIN	20 Low	pg	(27-31)	Final	DAYTON, OH VAMC
ERYTHROCYTE MEAN CORPUSCULAR HEMOGLOBIN CONCENTRATION	30 Low	gm/dl	(33-37)	Final	DAYTON, OH VAMC
ERYTHROCYTES	4.44 Low	m/cmm	(4.7-6.1)	Final	DAYTON, OH VAMC
HEMATOCRIT	47.1	%	(42-52)	Final	DAYTON, OH VAMC
HEMOGLOBIN	15.5	g/dl	(14-18)	Final	DAYTON, OH VAMC
LEUKOCYTES	20 High	t/cmm	(4.8-10.8)	Final	DAYTON, OH VAMC
LYMPHOCYTES/100 LEUKOCYTES	14.9 Low	%	(20.5-51.1)	Final	DAYTON, OH VAMC
MEAN CORPUSCULAR VOLUME	100.2 High	fl	(80-94)	Final	DAYTON, OH VAMC
PLATELET MEAN VOLUME	60 High	fl	(7.4-10.4)	Final	DAYTON, OH VAMC
PLATELETS	50 Low	t/cmm	(130-400)	Final	DAYTON, OH VAMC

Comments: This is a comment entered for this lab panel.

Performing Location Name/Address:

DAYTON, OH VAMC 4100 W. THIRD STREET , DAYTON, OH 45428

Lab Test:	Carbon Dioxide Content		
Lab Type:	Chemistry/Hematology	Ordering Provider:	XXXXXX
Specimen:	Serum	Ordering Location:	DAYTON, OH VAMC
Date/Time Collected:	01 Dec 2010		

Test Name	Result	Units	Reference Range	Status	Performing Location
CARBON DIOXIDE		mEq/L	(23-33)	Pending	DAYTON, OH VAMC

MEDICAL CONFIDENTIAL

Labs & Tests

Source:	Self-Entered

Test Name:	Blood Test	Date:	06 Jun 2010
Location Performed:	Community Center	Provider:	Red Cross Blood Drive
Results:	Was not able to donate blood because iron was low		
Comments:	Will ask doctor at next visit		

Test Name:	Colonoscopy	Date:	01 Jul 2010
Location Performed:	VAMC	Provider:	Provider One
Results:	No new polyps		
Comments:	Keep high fiber diet		

MEDICAL CONFIDENTIAL
Blue Button Download My Data SM

Vitals & Readings

Source:	Self-Entered

Measurement Type:	Blood pressure	Date:	02 Aug 2010
Systolic:	130	Time:	1720
Diastolic:	76		
Comments:	BP taken lying down		

Measurement Type:	Blood pressure	Date:	02 Aug 2010
Systolic:	132	Time:	1730
Diastolic:	76		
Comments:	BP taken standing. BP continues at goal. Doctor says to continue BP medications as directed		

Measurement Type:	Heart rate	Date:	02 Jun 2010
Heart Rate:	160	Time:	1720
Comments:	Started taking Beta-Blockers after visit with physician		

Measurement Type:	Body weight	Date:	02 Apr 2010
Body Weight:	246	Time:	1720
Measure:	Pounds		
Comments:	Talk to provider about weight management program at next visit		

Measurement Type:	Body weight	Date:	02 May 2010
Body Weight:	244	Time:	1720
Measure:	Pounds		
Comments:	Lost a few pounds and feel better. Walking daily		

Measurement Type:	Body weight	Date:	02 Jun 2010
Body Weight:	242	Time:	1720
Measure:	Pounds		
Comments:	still walking when I have time off from work		

Measurement Type:	Body temperature	Date:	02 Mar 2010
Body Temperature:	98.5	Time:	1720
Measure:	Fahrenheit		
Method:	Mouth		
Comments:	I wasn't feeling well but temperature is normal		

Measurement Type:	Pain	Date:	01 Jan 2010
Pain Level:	7	Time:	1720
Comments:	Lower back pain - took 1 Ibuprofen with food for pain		

MEDICAL CONFIDENTIAL

Family Health History

Source:	Self-Entered

Relationship:	Self
First Name:	ONE
Last Name:	MHVVETERAN
Living or Deceased	Living
Health Issues:	Back Pain<br>Insomnia<br>>1 beer/wine a day<br>Hearing Loss<br>Pneumonia<br>Smoking >1 pack/day<br>Allergies<br>Chicken Pox<br>Current Smoker<br>Diabetics Type 2<br>Overweight<br>High Blood Pressure<br>Depression<br>High Blood Cholesterol<br>Stomach/Bowel Other<br>Smoking >20 Years
Other Health Issues:	trouble sleeping
Comments:	I sometimes have trouble sleeping when stress is high at work

Relationship:	Mother
First Name:	Four
Last Name:	MHVVeteranMother
Living or Deceased	Deceased
Health Issues:	Cancer Other<br>Diabetics Type 2<br>Overweight<br>Joint Pain<br>Stroke
Other Health Issues:	Chronic joint pain
Comments:	Mother died of cancer at age 40

MEDICAL CONFIDENTIAL

Military Health History

**Source:**	Self-Entered

**Event Title:**	Overseas Deployment
**Event Date:**	7 Apr 2002
**Service Branch:**	Army
**Rank:**	Captain
**Exposures:**	Yes
**Location of Service:**	Overseas
**Onboard Ship:**	No
**Military Occupational Specialty:**	Infantry
**Assignment:**	1st Recon
**Exposures:**	Iraq: Exposed to burning chemicals
**Military Service Description:**	Unit was in charge of security

END MY HEALTHEVET PERSONAL HEALTH INFORMATION

MEDICAL CONFIDENTIAL
My healthevet
Blue Button Download My Data SM
